# Metabolic Syndrome May Not be a Good Predictor of Coronary Artery Disease in the Iranian Population: Population-Specific Definitions are Required

**DOI:** 10.1100/tsw.2009.17

**Published:** 2009-02-15

**Authors:** Mahmoud Ebrahimi, Seyyed Mohammad Reza Kazemi-Bajestani, Majid Ghayour-Mobarhan, Mohsen Moohebati, Roghaye Paydar, Mohsen Azimi-Nezhad, Habib Ollah Esmaily, Gordon A. A. Ferns

**Affiliations:** ^1^ Cardiovascular Research Center, Avicenna (Bu-Ali) Research Institute, Mashhad University of Medical Science (MUMS), Mashhad, Islamic Republic of Iran; ^2^ Atherosclerosis Research Center, Avicenna (Bu-Ali) Research Institute, MUMS, Mashhad, Islamic Republic of Iran; ^3^ Department of Cardiology, Faculty of Medicine, MUMS, Mashhad, Islamic Republic of Iran; ^4^ Department of Nutrition and Biochemistry, Faculty of Medicine, MUMS, Mashhad, Islamic Republic of Iran; ^5^ Department of Biostatistics, Faculty of Medicine, MUMS, Mashhad, Islamic Republic of Iran; ^6^ Postgraduate Medical School, University of Surrey, Stag Hill, Guildford, Surrey, UK

**Keywords:** metabolic syndrome, coronary artery disease, angiography

## Abstract

Metabolic syndrome (MS) is associated with an increased risk of coronary artery disease (CAD) in Western populations. We have investigated the relationship between the presence of MS and other conventional risk factors, and angiographically defined CAD, in a Middle Eastern population. Patients (n = 431) attending a hospital cardiology clinic for angiography were assessed. Each patient subsequently underwent routine angiography. Anthropometric and biochemical data were used to establish whether patients had MS, using either IDF or NCEP-ATP III criteria. The relationship between the presence of MS, or other individual coronary risk factors, and angiographically defined CAD was assessed by logistic regression analysis. A further reference group of individuals without overt CAD (n = 1276) was used as an additional comparator group. Of the 431 patients, 327 (75.9%) were found to have angiographically defined CAD. There was no significant relationship between MS, using either the IDF or NCEP-ATP III definitions, and CAD in this population. Of the parameters assessed, age, total cholesterol, and low serum HDL cholesterol were the strongest independent predictors of angiographically defined CAD (*p* < 0.01, *p* < 0.01, *p* < 0.05, respectively). It appears that within an Iranian population, the presence of MS defined by either the NCEP-ATP III or IDF criteria fails to identify individuals with established, angiographically defined CAD. However, a low serum HDL cholesterol, a component of MS, was an important independent predictor of CAD in this population. It is possible that the criteria for defining MS as a risk predictor of CAD in Middle Eastern populations may need to be revised.

### INTRODUCTION

The metabolic syndrome (MS) is characterized by a clustering of clinical and metabolic features that include high triglycerides, low HDL cholesterol, high blood pressure, impaired glucose tolerance, visceral adiposity, and insulin resistance[1,2,3]. This clustering of cardiovascular risk factors led to the notion of a syndrome that has been variously called syndrome X, MS, Reaven's syndrome, and insulin resistance syndrome[4,5].

The World Health Organization (WHO) initially proposed a definition for MS in 1998[6]. The Third Report of the National Cholesterol Education Program (NCEP) Expert Panel on Detection, Evaluation, and Treatment of High Blood Cholesterol in Adults (Adult Treatment Panel III [ATP III]) provided a new working definition of MS[2]. A further definition of MS has recently been proposed by the International Diabetic Federation (IDF) in which the importance of central adiposity has been stressed and different cut-off criteria are applied to waist circumference measurements in different ethnic groups[7]. Because of the lack of consensus on the criteria for the definition of MS, its estimated prevalence has varied markedly between different studies[8,9]. According to the NCEP-ATP III definition, it is about 22% in the U.S.[10].

Individuals with MS are at high risk of coronary artery disease (CAD)[1,2,3]. CAD is highly prevalent and is the single greatest cause of mortality in Iran[11,12]. MS is also very prevalent in Iran. The unadjusted prevalence of MS in an urban population of Iran (from Tehran) was 30.1% and age-standardized prevalence was 33.7%[13].

In the present study, we wished to investigate the relationship between the presence of MS (using the NCEP-ATP III and IDF definitions) and its constituent factors, and angiographically defined CAD, in an Iranian population sample.

### MATERIALS AND METHODS

#### Subjects

The study was carried out on a sequential sample of 1707 subjects (832 males and 875 females). Subjects (n = 431) were candidates for routine angiography according to standard indications and underwent coronary angiography at the Ghaem Medical Education Hospital, Mashhad, Iran. According to the results of angiography, these latter individuals could be divided into two groups: those with angiographically defined CAD[CAD(+)] (case group) and those with a normal angiogram [CAD(–)] (control group). The remaining 1276 subjects were drawn from the same local population base as the patients, but were not suspected of having CAD; therefore were not eligible for angioplasty and were used as a reference group. Ghaem Hospital is a governmental hospital without private rooms, so patients are not selected on the basis of socioeconomic status. None of the subjects had a past history of coronary angioplasty or coronary artery bypass graft (CABG). They ranged in age from 33 to 80 years. Patients who were on lipid-lowering medication, oral contraceptives, or hormone replacement therapy were excluded from the study.

Each patient gave informed written consent to participate in the study, which was approved by the Mashhad University of Medical Science Ethics Committee.

#### Anthropometric Measurement

For all individuals, anthropometric parameters including weight, height, and waist circumference were measured using standard protocols. Height, body weight, and waist circumference were measured with subjects dressed in very light clothing after an overnight fast. Waist circumference was measured at the level of the umbilicus (at the level midway between the lower rib margin and the iliac crest). Body weight was measured with a standard scale to an accuracy of ±0.1 kg and height was measured to an accuracy of ±0.1 cm (a stadiometer was used for measuring height). Blood pressure (BP) was measured twice while patients were seated and rested for 15 min, using a standard mercury sphygmomanometer calibrated by the Iranian Institute of Standards and Industrial Research. The interval between each BP measurement was at least 30 min, and in this interval patients were at rest (not doing heavy activities or running) and the average of the two measurements was taken as the BP. The systolic BP was defined as the appearance of the first sound (Korotkoff phase 1) and the diastolic BP was defined as the disappearance of the sound (Korotkoff phase 5) during deflating of the cuff. Body mass index (BMI) was calculated as weight (kg) divided by height squared ($m^2$).

### Angiographic Assessment

Coronary angiograms were performed using routine procedures. Analysis of the angiograms was performed offline by a specialist cardiologist. The presence of one or more stenoses ≥50% in diameter of at least one major coronary artery (left main, right coronary artery, left anterior descending, circumflex) was considered evidence of significant CAD [CAD(+)][14]. Patients in whom stenoses ≥50% in diameter could not be identified were considered to have a normal angiogram [CAD(–)].

### Routine Biochemical Analysis

A full fasted lipid profile, comprising total cholesterol, triglycerides, high-density lipoprotein cholesterol (HDL-C), and low-density lipoprotein cholesterol (LDL-C), was determined for each patient. Serum lipid and fasting blood sugar (FBS) concentrations were measured by enzymatic methods.

### Applied Definitions

Two standard definitions of MS were applied in our study (NCEP-ATP III and IDF). The criteria of each definition are summarized below.

**NCEP-ATP III**[2] — The latter was defined as being present when three of the following criteria were met:

- Increased waist circumference: 102 cm (40 in.) for men and 90 cm (35 in.) for women
- Low plasma level of HDL-C: 1.04 mmol/l (40 mg/dl) for men and 1.30 mmol/l (50 mg/dl) for women
- Increased values for plasma triglycerides: 1.70 mmol/l (150 mg/dl)
- Elevated BP: ≥130/85 mmHg
- Elevated level of blood sugar: 6.11 mmol/l (110 mg/dl)

**IDF**[7] — According to the IDF definition, MS is present in persons having central obesity (defined as a waist circumference of at least 94 cm in men or 80 cm in women) and meeting at least two of the following criteria:

- Fasting serum triglycerides: ≥1.70 mmol/l (150 mg/dl)
- HDL-C: 1.04 mmol/l (40 mg/dl) in men or 1.30 mmol/l (50 mg/dl) in women
- BP: ≥130/85 mmHg
- Fasting glucose: ≥6.11 mmol/l (100 mg/dl)

### Reference Group

There were 1276 subjects with no previous history of any major disease including malignancies, cardiovascular, metabolic, or infective diseases. The general assessment of inclusion of subjects in this group was based on physical examination and completion of the past medical history forms by the volunteers. Volunteers were recruited from normal populations by advertisements. Each volunteer gave informed written consent to participate in the study, which was approved by the Mashhad University of Medical Science Ethics Committee. All the laboratory analysis of the control group was performed in free status for the subjects.

### Statistical Analysis

The data were subjected to statistical evaluation using Mini-Tab (release 13, Minitab Inc., 2000, State College, PA), with descriptive statistics (mean, median, standard deviation [SD], and interquartile range) being determined for all variables. Data were assessed for normality using the Kolomogorov-Smirnov test. In our comparisons, t-tests and chi-square tests were used for quantitative and qualitative variables using a Bonferoni correction for multiple comparisons. Analysis of covariance (ANCOVA) was used to assess differences after adjustment for important confounding factors including age, gender, smoking habit, BP, FBS, lipid profile, and BMI. Logistic regression analysis was used to predict whether CAD is related to metabolic and traditional coronary risk factors. The variables that were analyzed in the regression model were continuous variables including age, waist circumference, triglycerides, LDL, total cholesterol, and HDL. To enable adjustment for potential confounding factors, we entered the factors of age, gender, BMI, smoking, FBS, lipid profile, systolic and diastolic BP, and MS into the equation. A $p < 0.05$ was considered significant. A person who had FBS ≥126 mg/dl[15] or had a prior diagnosis of diabetes, or used antidiabetic medication was considered to be diabetic. A person whose BP was more than 140/90 mmHg[16] or used antihypertensive medications was considered to be hypertensive.

## RESULTS

### Demographic Characteristics and the Presence of MS in Males and Females

The mean age of subjects was 56.7 ± 10.9 years. Among the whole group, 832 (48.7%) were male and 875 (52.3%) were female (Table 1).

Of the patients, 327 (75.9%) were CAD(+) and 104 (24.1%) subjects were CAD(–). Among the 327 subjects who were CAD(+), 206 (63.0%) were male and 121 (27.0%) were female. Of the 104 subjects who were CAD(–), 38 (36.5%) were male and 66 (73.5%) were female (Table 1).

Using the IDF definition, 827 (48.4%) of the total group of 1707 subjects had MS (MSIDF) and 776 (45.5%) had MS using the NCEP-ATP III criteria (MSATP III). The proportion of females who had MS within the whole group was significantly higher compared to males using either definition of MS; MSIDF ($p < 0.001$, Table 1) or MSATP III ($p < 0.001$, Table 1).

### Age and Smoking Status in Angiographically Defined CAD With and Without MS

The age and smoking status of CAD(+) patients with and without MS using either definition were not statistically different ($p > 0.05$, Table 2).

**TABLE 1**
**Clinical and Biochemical Characteristics of Patients With and Without Angiographically Defined CAD**

| | CAD(+) n = 327 | CAD(–) n = 104 | Reference Group n = 1276 |
|---|---|---|---|
| Age (years) | 56.51 ± 10.64 | 51.48 ± 10.98 | 52.75 ± 7.05 |
| Male/female | 206/121 | 38/66 | 588/687 |
| Smokers | 99 (30.5%) | 22 (21.2%) | 308 (24.1%) |
| Waist circumference (cm) | 96.43 ± 12.76* | 93.97 ± 16.12 | 92.23 ± 14.71 |
| BMI (kg/m$^2$) | 26.49 ± 5.56 | 27.11 ± 4.55 | 25.92 ± 4.98 |
| Systolic BP (mmHg) | 129.13 ± 22.48 | 128.58 ± 16.49 | 134.48 ± 22.63 |
| Diastolic BP (mmHg) | 80.15 ± 12.16 | 78.64 ± 10.73 | 83.56 ± 13.85 |
| FBS (mg/dl) | 119.75 ± 75.45 | 98.83 ± 22.74 | 93.88 ± 44.73 |
| TG (mg/dl) | 117 (93.25–162.75)** | 129 (86–173)** | 145 (97–216) |
| LDL-C (mg/dl) | 132.60 ± 42.09** | 122.65 ± 35.81 | 114.38 ± 43.86 |
| Total cholesterol (mg/dl) | 205.74 ± 50.58** | 196.47 ± 40.14 | 184.82 ± 48.49 |
| HDL-C (mg/dl) | 48.42 ± 11.79** | 47.55 ± 8.60** | 32.80 ± 13.80 |
| Obesity (BMI > 30) | 66 (20.7%) | 28 (27.2%) | 246 (19.3%) |
| DM | 82 (25.2%)** | 6 (5.8%) | 98 (7.7%) |
| Hypertensive | 235 (72.5%)* | 64 (62.1%) | 804 (63%) |
| MSIDF | 118 (37.9%) | 48 (50%) | 661 (51.8%) |
| Male | 50 (25.38%) | 7 (19.44%) | 191 (32.5%) |
| Female | 68 (59.65%) | 41 (68.33%) | 470 (68.3%) |
| NCEP-ATP III | 94 (31.4%) | 36 (39.1%) | 646 (50.60%) |
| Male | 37 (19.80%) | 3 (9.09%) | 203 (34.5%) |
| Female | 57 (50.90%) | 33 (55.93%) | 443 (64.4%) |

Values expressed as mean ± SD for normally distributed data, and median and interquartile range for non-normally distributed data. Between groups comparisons were assessed by parametric statistical analysis for normal distributed data and nonparametric test for nondistributed data. CAD(+), subjects with angiographically defined CAD; CAD(–), subjects without angiographically defined CAD; MSIDF, MS based on International Diabetic Federation definition; NCEP-ATP III, MS based on Adult Treatment Panel III definition; BMI, body mass index; FBS, fasting blood sugar; HDL, high-density lipoprotein; LDL, low-density lipoprotein; DM, diabetes mellitus; TG, triglyceride. Compared with reference group; *$p < 0.05$, **$p < 0.001$.

### Comparisons of Metabolic Risk Factors in CAD(+) and CAD(–) Groups

The group of CAD(+) patients was approximately 4.5 years older than the CAD(–)and reference groups ($p = 0.05$, Table 1). The proportion of current smokers did not differ between the CAD(–) and CAD(+) groups and control group ($p > 0.05$, Table 1). Indices of adiposity were higher in the CAD(+) group, with waist circumference significantly greater than that for the CAD(–) ($p = 0.05$, Table 1) and control groups; however, BMI and presence of obesity (BMI > 30 kg/m$^2$) did not differ between the three groups ($p > 0.05$ for three, Table 1).

FBS was significantly higher in the CAD(+) group ($p < 0.001$, Table 1) and BP was not significantly different between the two groups ($p > 0.05$, Table 1).

**TABLE 2**
**Demographic Characteristics of Patients with MS Defined by IDF and ATP III Criteria**

| | IDF | ATP III |
|---|---|---|
| Gender | | |
| Male | 64 (24.2%)* | 43 (17.5%)** |
| Female | 123 (63.4%) | 105 (55.6%) |
| Smoking | | |
| Yes | 62 (52.6%) | 38 (60.2%) |
| No | 56 (47.4%) | 56 (59.8%) |
| Age | 54.97 ± 9.63 | 55.85 ± 9.49 |

Values expressed as mean ± SD; IDF, MS based on International Diabetic Federation definition; ATP III, MS based on Adult Treatment Panel III definition; between groups comparisons were assessed by chi-square or t-test; *$p < 0.001$, **$p < 0.01$.

Serum fasting HDL ($p < 0.05$), LDL ($p = 0.05$), and total cholesterol concentrations ($p < 0.001$) were significantly higher for the CAD(+) group compared to the CAD(–) and reference groups ($p < 0.001$, Table 1), while serum triglyceride ($p < 0.05$) concentrations were significantly lower in the CAD(+) group compared to the CAD(–) and reference groups ($p < 0.05$, Table 1). These differences between the CAD(–) and CAD(+) groups remained significant after adjusting for confounding factors.

The proportion of hypertensive and diabetic patients did differ between the CAD(+) and other groups (Table 1). Statistical comparison of the proportion of subjects taking either antidiabetic or antihypertensive medication did not differ between the three groups ($p > 0.05$, Table 1).

### MS in the CAD(–) and CAD(+) Groups

The proportion of patients with MS, defined by either the IDF or NCEP-ATP III, was not significantly different between three groups ($p > 0.05$, Table 1).

### Logistic Regression Analysis of Different Associated Factors with CAD

Multiple logistic regression analysis was performed to assess the relative importance of selected parameters (age; waist circumference; triglycerides; LDL, total, and HDL cholesterol; and MS) in determining the presence of angiographically defined CAD (Table 3). Age, total cholesterol, and HDL proved to be significant predictors of CAD ($p < 0.001$, $p < 0.001$, $p < 0.01$, respectively), with age being the strongest predictor. The presence of MS was not a predictor of CAD in this population. This logistic regression analysis was performed for the patients with angiographically defined CAD.

## DISCUSSION

### Association Between the Presence of MS and CAD in Iranian Subjects

MS is characterized by the presence of a number of established coronary risk factors and has been shown to be related to an increased risk of cardiovascular and microvascular events in several populations[9,17, 18,19,20,21,22,23,24]. In this present study, we found no statistically significant relationship between the

**TABLE 3**
**The Relative Risk of Angiographically Defined CAD Associated with Individual Coronary Risk Factors**

| Variable | Odds Ratio (95% CI) |
|---|---|
| Age (year) | 1.05 (1.02–1.07)* |
| TG (mg/dl) | 1.002 (1.000–1.004) |
| Total cholesterol (mg/dl) | 0.99 (0.98–0.99)* |
| HDL cholesterol (mg/dl) | 1.11 (1.09–1.12)** |

Adjusted odds ratios with 95% confidence intervals (95% CI) obtained from multiple logistic regression. Models were adjusted by logistic regression analysis for the association with CAD among our subjects. TG, triglyceride; HDL, high density lipoprotein; *$p < 0.001$, **$p < 0.01$.

presence of MS and angiographically defined CAD. There may be several reasons for this lack of association in this particular Iranian population. It is possible that the criteria used for defining MS in a Caucasian population may be inappropriate for an Iranian population, as has been found to be the case in other ethnic groups[25]. It may be necessary to modify the cut-off values for waist circumference and HDL cholesterol, and this will require further population-based studies. Screening by physicians prior to referral for angiography may have led to a higher proportion of subjects with MS being referred who did not have CAD. This would lessen the predictive power of the presence of MS. Although coronary angiography is the gold standard method for visualizing occlusive coronary disease, the culprit lesion responsible for an acute coronary event may often be associated with <50% occlusion by angiography[26]. While it is not possible to be entirely certain that all subjects within the control and reference groups were free of CAD, they did not have significant clinical deficit.

However, we have previously shown that levels of hs-CRP are raised in patients with MS[27] and it is therefore possible that individuals who do not have CAD (>50% stenosis) will nevertheless have a coronary event. A cohort study design would be necessary to confirm this.

### Gender and MS

Within our group of subjects referred for angiography, the proportion of females with MS was significantly higher compared to males. This is in accordance with the findings of several reports for the prevalence of MS in unselected population studies[28,29,30], including a study in Iran[13]. However, in recent years, it appears that the prevalence of MS among men is increasing[31,32].

### Effect of Age, Sex, and Smoking on CAD

We found a positive relationship between age and the presence of angiographically defined CAD. These findings are unsurprising given the progressive nature of atherosclerosis with time, and are similar to several previous reports[33,34,35].

Consistent gender differences in the prevalence, morbidity, and mortality associated with coronary disease has been reported[36,37,38]. In our study, we did not find a significant relationship between gender and the presence of angiographically defined CAD. Approximately 62% of women and 73% of men referred for angiography were found to be positive for CAD. Again, this is most likely to be due to the selection of patients referred for angiography and not a reflection of the prevalence of CAD among men and women in the general population. The relationship between smoking and CAD is well established[39,40], but although the proportion of patients positive for CAD who were smokers was higher than for patients without angiographically defined CAD, this failed to reach statistical significance in this sample.

### Diabetes Mellitus, Hypertension, and CAD

The frequency of diabetes and hypertension was found to differ significantly between the CAD(–) and CAD(+) groups. The prevalence of both conditions is high and increasing in the Middle East, and in our population, a large proportion of subjects had one or both conditions. This was the case in patients with or without angiographically defined CAD. The high prevalence of hypertension in the normal population possibly reflects the high overall prevalence in the general population in Iran and the fact that screening for hypertension is strongly recommended for the Iranian population by their physicians, especially after 50 years of age.

### Adiposity and CAD

Although the proportion of patients who were obese, and the mean BMI was greater in the CAD(+) group, this failed to reach statistical significance. Nevertheless, the mean waist circumference of these subjects was found to be significantly higher compared to the CAD(–) group, independent of gender. The relationship between adiposity and CAD is well established[41]. This may be linked to the increased risk of glucose intolerance, insulin resistance, and diabetes among obese individuals, and waist circumference is reported to the best predictor of insulin resistance[42].

### Lipid Profile and CAD

We found a positive association between serum total cholesterol, LDL cholesterol, and triglycerides with the presence of CAD, while serum HDL cholesterol concentrations were inversely related to CAD. Logistic regression analysis showed that a low serum HDL and high total cholesterol were the most important lipid-related determinants of CAD in our population. We also found that serum triglycerides were significantly higher in the subjects with CAD. A meta-analysis of 17 prospective trials found hypertriglyceridemia to be an independent risk factor for cardiovascular disease[43]. An increase in the number of atherogenic, small, dense LDL particles may arise from a defect in the metabolism of triglyceride-rich lipoproteins[44]. Although serum triglyceride concentration did not appear to be an independent risk factor for CAD following logistic regression analysis, this is probably due to the strong inverse relationship between serum triglycerides and HDL cholesterol that has been reported previously[45].

A low serum HDL concentration was found to be one of the strongest factors that independently associated with CAD in our population. HDL is thought to be involved in reverse cholesterol transport[46] and may also inhibit LDL oxidation[47]. The relationship between low serum HDL cholesterol and an increased risk of cardiovascular disease is well established in Caucasian populations[48,49,50,51,52,53,54,55].

### Limitations of the Current Study

The present study has certain limitations that need to be taken into account when considering the study and its contributions. The numbers of case and control groups are fewer than the reference group and therefore may result in some bias. The socioeconomic status and dietary intake were not considered in this study, and both may be important determinants of CAD risk in the Iranian population.

### CONCLUSION

Components of MS (central obesity, hypertension, glucose intolerance, low HDL, and high triglycerides) are common among patients with CAD, but the current definitions of MS do not allow adequate discrimination between individuals with and without angiographically defined CAD. Definitions of MS may need to be derived that take ethnogeographical factors into consideration. The impact of other risk factors, e.g., LDL cholesterol and smoking status, are clearly important when assessing overall risk in these populations. It may be necessary to redefine the cut-off criteria for MS for Middle Eastern populations, as they have been for some other racial groups.

Our results underscore the importance of low serum HDL cholesterol as a determinant of CAD in a Middle Eastern population.

### ACKNOWLEDGMENTS

This research project has been supported by the Mashhad University of Medical Science Research Council. We express appreciation to the staff of the Cardiology Department of Ghaem Hospital. The participation of the staff of Bu-Ali Research Institute of the Mashhad University of Medical Science is also gratefully acknowledged.
